# Characterization of a New *Vaccinia virus* Isolate Reveals the C23L Gene as a Putative Genetic Marker for Autochthonous Group 1 Brazilian *Vaccinia virus*

**DOI:** 10.1371/journal.pone.0050413

**Published:** 2012-11-26

**Authors:** Felipe L. Assis, Gabriel M. F. Almeida, Danilo B. Oliveira, Ana P. M. Franco-Luiz, Rafael K. Campos, Maria I. M. Guedes, Flávio G. Fonseca, Giliane S. Trindade, Betânia P. Drumond, Erna G. Kroon, Jônatas S. Abrahão

**Affiliations:** 1 Departamento de Microbiologia, Universidade Federal de Minas Gerais, Belo Horizonte, Minas Gerais, Brazil; 2 Departamento de Medicina Veterinária Preventiva, Universidade Federal de Minas Gerais, Belo Horizonte, Minas Gerais, Brazil; 3 Departamento de Parasitologia, Microbiologia e Imunologia, Universidade Federal de Juiz de Fora, Juiz de Fora, Minas Gerais, Brazil; Instituto de Higiene e Medicina Tropical, Portugal

## Abstract

Since 1999, several *Vaccinia virus* (VACV) isolates, the etiological agents of bovine vaccinia (BV), have been frequently isolated and characterized with various biological and molecular methods. The results from these approaches have grouped these VACV isolates into two different clusters. This dichotomy has elicited debates surrounding the origin of the Brazilian VACV and its epidemiological significance. To ascertain vital information to settle these debates, we and other research groups have made efforts to identify molecular markers to discriminate VACV from other viruses of the genus *Orthopoxvirus* (OPV) and other VACV-BR groups. In this way, some genes have been identified as useful markers to discriminate between the VACV-BR groups. However, new markers are needed to infer ancestry and to correlate each sample or group with its unique epidemiological and biological features. The aims of this work were to characterize a new VACV isolate (VACV DMTV-2005) molecularly and biologically using conserved and non-conserved gene analyses for phylogenetic inference and to search for new genes that would elucidate the VACV-BR dichotomy. The VACV DMTV-2005 isolate reported in this study is biologically and phylogenetically clustered with other strains of Group 1 VACV-BR, the most prevalent VACV group that was isolated during the bovine vaccinia outbreaks in Brazil. Sequence analysis of C23L, the gene that encodes for the CC-chemokine-binding protein, revealed a ten-nucleotide deletion, which is a new Group 1 Brazilian VACV genetic marker. This deletion in the C23L open reading frame produces a premature stop-codon that is shared by all Group 1 VACV-BR strains and may also reflect the VACV-BR dichotomy; the deletion can also be considered to be a putative genetic marker for non-virulent Brazilian VACV isolates and may be used for the detection and molecular characterization of new isolates.

## Introduction

The smallpox eradication campaign, which was promoted by the World Health Organization (WHO) in the 1960s and 1970s, represents a major milestone in medical history [1], [2]. After centuries of epidemics and deaths, smallpox was declared eradicated in 1980. The etiological agent of smallpox is the *Variola virus* (VARV), a member of the *Poxviridae* family, which comprises other virus species that are associated with mild to severe exanthematic diseases in a broad host-range [1]. The smallpox vaccines used in the WHO campaign were, in fact, strains of the *Vaccinia virus* (VACV), a species belonging to the genus *Orthopoxvirus* (OPV), which induced serological cross-reactivity against other OPV members, including VARV [3], [1]. With smallpox eradicated, smallpox vaccination was suspended due to several cases of adverse manifestations from the vaccine [4].

Despite this remarkable victory against VARV, the suspension of smallpox vaccination led to the emergence of a generation that is susceptible to other OPV species [4]. This fact may explain the emergence of zoonotic OPV species such as *Cowpox virus* (CPXV) in Europe [5]; *Monkeypox virus* (MPXV) [6], which occurs naturally in Africa and was recently introduced in the USA; and, ironically, VACV in rural areas of Brazil and India, which has been associated with exanthematic outbreaks in both humans and cattle [7], [8], [9]. Although some authors believed that VACV vaccine strains could have spread from humans to domestic animals and adapted to the rural environment, other studies have suggested an independent origin for the South American VACV isolates, which are distinct from the vaccine strains used on this continent during the WHO campaign [10], [11]. Nevertheless, Brazilian VACV (VACR-BR) strains may have more than one origin, vaccinal or autochthonous. Regardless of its origins, the VACV strains have proven to be well-adapted to Brazilian rural and wild environments and have been detected in bovines, humans, rodents, monkeys, horses and other vertebrate species [8], [12], [13], [14].

Several VACV outbreaks in Brazil, which resulted in economic losses and had public health impacts, have been described since 1999 [7], [8], [15], [16], [17]. During these outbreaks, infected dairy cattle usually presented ulcerative lesions on their teats and udders and had decreased milk production [8], [15], [16], [17]. Rural workers who were infected with VACV, most likely from occupational contact with infected cattle, usually presented lesions on their hands and arms, lymphadenopathy, high fever and prostration, among other symptoms [18]. The introduction and spread of VACV between farms are usually linked to cow milking and cattle trade [12]. Since early reports of VACV outbreaks in Brazil, dozens of VACV isolates have been characterized [7], [8], [15], [16], [17]. Molecular studies have shown that Brazilian VACV strains can be divided into two distinct groups: Group 1 and Group 2 [10], [11]. The Group 1 VACV-BR comprises Cantagalo, Araçatuba, Passatempo, GuaraniP2, Mariana, Pelotas2 and other strains; Group 2 VACV-BR includes GuaraniP1, Pelotas1, Bean58058 and other strains [10], [11], [14]. Interestingly, this molecular dichotomy is also reflected in certain biological properties of the strains, including virulence in the BALB/c mouse model and plaque phenotype in BSC-40 cells [12], [14], [19]. Although each VACV strain possesses unique genetic characteristics, most of them are very similar to each other within the same group, especially those belonging to Group 1; they most likely share a common ancestor. Nevertheless, our group and others have assigned specific designations for each newly discovered isolate, which refer to the unique characteristics of each outbreak.

Studies of Brazilian VACV have advanced our knowledge in the past few years, and some genes were identified as molecular markers to differentiate VACV from other OPV members or to discriminate between the Brazilian VACV groups. The viral growth factor gene (*vgf*) has been a marker that is screened by PCR because it is conserved among OPV members and duplicated in most OPV genomes [20]. However, *vgf* sequencing only allows for VACV species identification but not for VACV strain differentiation. Other conserved genes had been sequenced from Brazilian VACV because *vgf* is not remarkably different between VACV groups 1 and 2 [20]. For the past few years, A56R has been the most widely used gene marker in genetic analyses for Brazilian VACV strain differentiation. Damaso et al. (2000) demonstrated that VACV Cantagalo strain (Group 1) has an 18-nucleotide (nt) deletion in A56R. Most of the new VACV isolates were analyzed for this gene. With the discovery of new VACV isolates, A56R proved to be even more interesting because some Group 2 isolates do not have this particular 18-nt deletion. Other molecular markers have been discovered in the past few years, such as the serine protease inhibitor-3 gene (K2L) and the A-type inclusion body gene (ATI), among others [11], [21], [22]. These approaches are important, not only for sample identification but also to infer ancestry and to investigate hypothetical correlation of each sample or group with its unique epidemiological and biological features. In this study, we described the isolation of a VACV that has been characterized by sequencing, along with five genes analyses that included traditional and new, conserved and variable genetic markers. Analysis of C23L, the gene that encodes the CC-chemokine-binding protein, revealed not only a new Brazilian VACV genetic marker but also a possible premature stop-codon mutation shared by Group 1 VACV strains.

**Figure 1:**
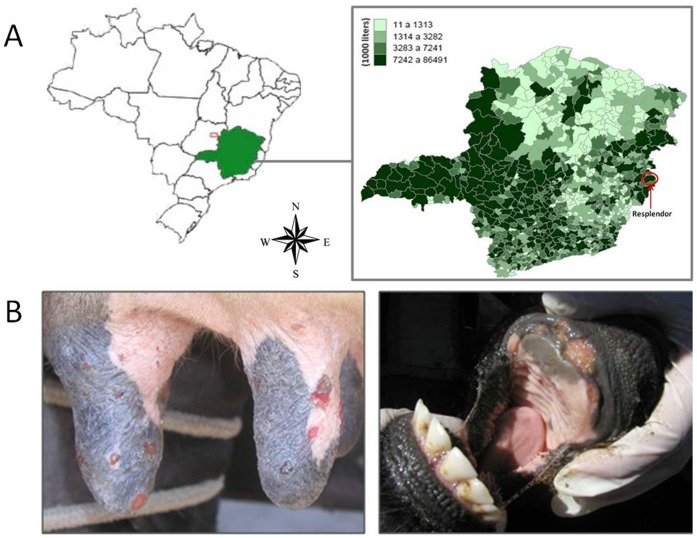
Map of the outbreak region and lesions found on cattle. (A) Overview map of Brazil (left) and the Minas Gerais State (right). The red circle highlights Resplendor County where the VACV DMTV-2005 strain was isolated. The expanded map shows the average milk production (liters) in each county. Resplendor is an important milk production region in the Minas Gerais State (governmental source/2006). (B) Lesions caused by DMTV infection. VACV-like lesions on the teats and udder of an infected dairy cow (left) and of the calf’s oral mucosa (right) are as shown.

## Materials and Methods

### Ethic Statement

This study was approved by the Comitê de Ética em Experimentação Animal - CETEA. In Brazil, it is not necessary to have specific ethics approval for collection of clinical samples from domestic cattle; all collection procedures were performed by veterinarians of a Brazilian Surveillance Institute (Instituto Mineiro de Agropecuária - IMA), following institutional recommendations (http://www.ima.mg.gov.br). Mice were sacrificed with anesthesia (ketamine + xilasin), followed by cervical dislocation.

(The animal experiments were performed before 1/12/2009.)

**Figure 2:**
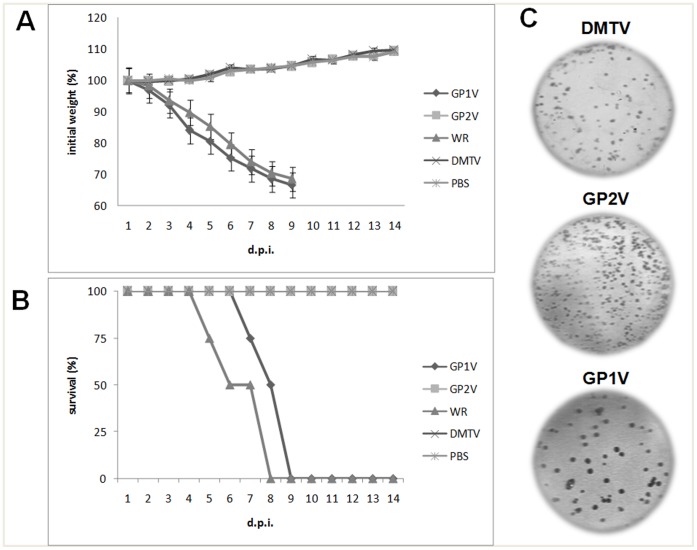
Infectivity assays of Brazilian VACV strains. (A) Body weight loss and (B) survival rates of BALB/c mice that were infected with VACV-BR. Groups of mice (n = 4) were inoculated intranasally with 10^6^ PFU of VACV. Weight and clinical signs of the mice were monitored daily (until day 14 p.i.), and animals that lost more than 25% of their initial weight were euthanized. The control group was inoculated with PBS. The error bars represent standard deviations. Both the weight loss and survival rates of the mice infected with the DMTV isolate were similar to those of GP2V-infected mice (C). Plaque phenotype assay on BSC-40, an epithelial kidney cell line. The results of the DMTV-2005, GP1V-(Group 2 control) and GP2V-infected (Group 1 control) samples are highlighted. DMTV-2005 exhibited small cytopathic effects similar to those of GP2V; GP1V showed larger plaques in the cell culture similar to those formed by the Group 2 Brazilian VACV.

### Cells and Viruses

African green monkey cells (BSC-40 cells; ATCC, USA) were grown at 37°C in Eagle’s Minimum Essential Medium (MEM) (GibcoBRL, Invitrogen, Carlsbad, California, USA), which was supplemented with 5% fetal calf serum (FCS) (Cultilab, Brazil), 25 mg/mL fungizone (Amphotericin B) (Cristália, São Paulo, Brazil), 500 U/mL penicillin and 50 mg/mL gentamicin (Schering-Plough, São Paulo, Brazil) and used for viral isolation [12].

**Figure 3:**
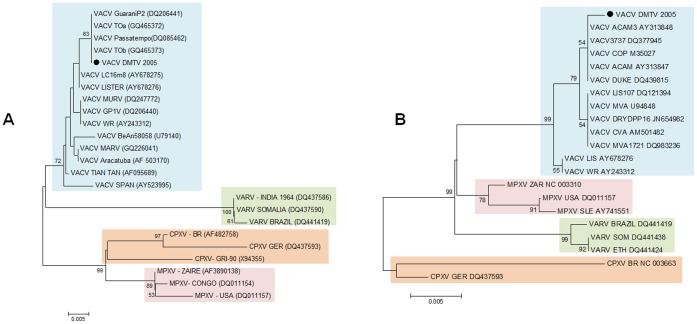
Phylogenetic trees generated from OPV nucleotide sequences, including VACV DMTV-2005. Phylogenetic analysis of the C11R (A) and H5R (B) sequences. The colored boxes are species-related (VACV, blue; VARV, green; CPXV, orange; and MPXV, pink). The OPV clusters (colored boxes) are strongly supported by high bootstrap values. The DMTV isolate is depicted as a black dot. Maximum likehood trees were reconstructed using diferent datasets containing sequences from the genes listed above, using MEGA 4.0. Using ModelTest, server [35] the nucleotide substitution model of Tamura 1992 was selected as the best one fitting the data. Rates of variation among sites were estimated for each dataset and two discrete Gamma categories were used to model evolutionary rate differences among sites and the reliability of branching patterns was tested through 1000 bootstrap sampling.

**Figure 4:**
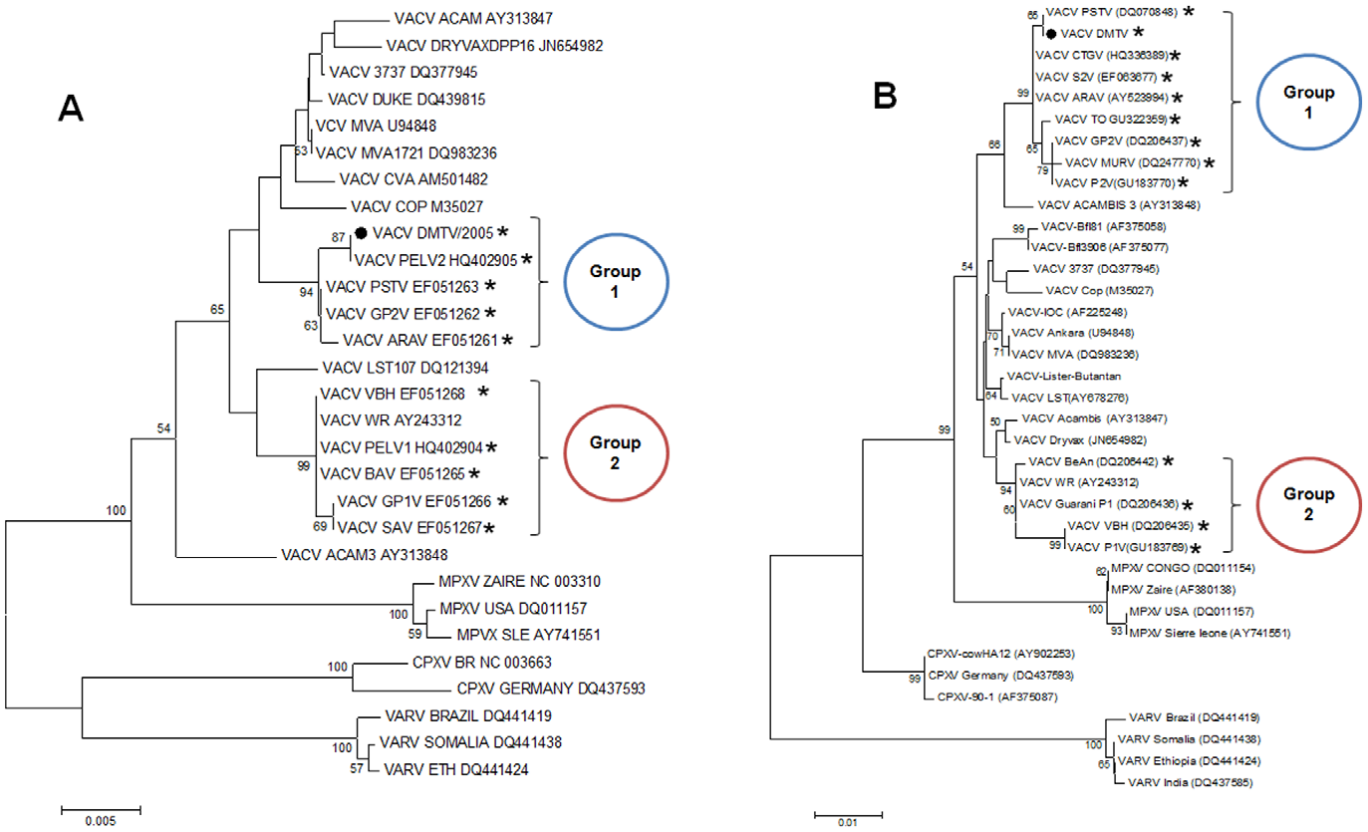
Phylogenetic trees generated from OPV nucleotide sequences, including the Brazilian VACV strains. Phylogenetic analyses of B5R (A) and A56R (B) sequences showed that the Brazilian VACV strains are split into two different branches (Groups 1 and 2), which do not cluster directly with the vaccine strains (vaccine strains: those with no asterisk; and VACV-WR, a laboratorial strain). The DMTV-2005 isolate is depicted as a black dot. The Brazilian VACV strains are depicted with asterisks. Maximum likehood trees were reconstructed using different datasets containing sequences from the genes listed above, using MEGA 4.0. Using ModelTest, server [35] the nucleotide substitution model of Tamura 1992 was selected as the best one fitting the data. Rates of variation among sites were estimated for each dataset and two discrete Gamma categories were used to model evolutionary rate differences among sites and the reliability of branching patterns was tested through 1000 bootstrap sampling.

VACV Western Reserve (VACV-WR) strain was kindly provided by Dr C. Jungwirth (Universitat Wurzburg, Germany) and used as a viral control in the biological assays. The VACV GuaraniP1 (GP1V) and GuaraniP2 (GP2V) strains were isolated by our team during an outbreak in 2001 [23] and are part of our biological collection. These and other Brazilian VACV strains were used as controls in the biological and molecular assays.

**Figure 5:**
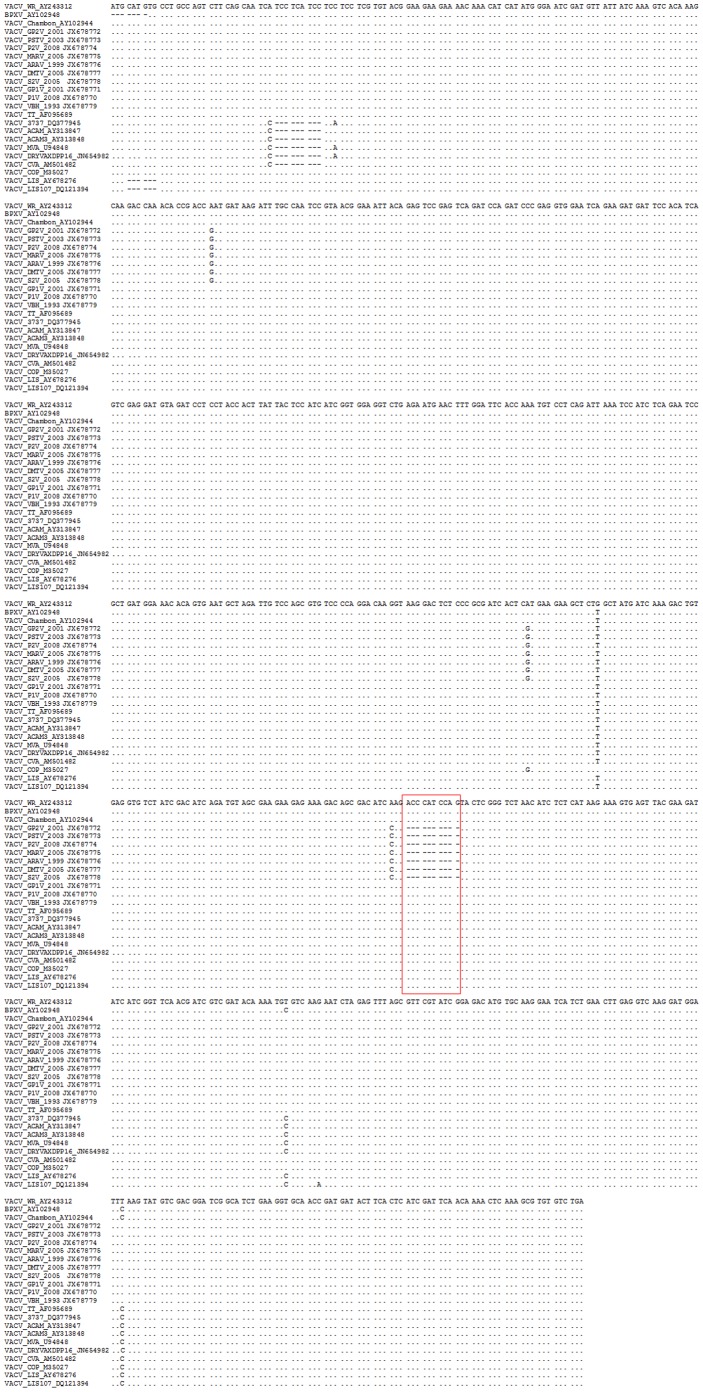
Nucleotide sequence alignment of C23L coding DNA sequences. Sequences were retrieved from GenBank and aligned using ClustalW method as implemented in the MEGA 4.0 program. The DMTV-2005 isolate showed a 10-nt deletion that leads to an early stop-codon and most likely results in non-functional protein production. The same deletion was observed in the Brazilian VACV Group 1 strains, including DMTV-2005. Nevertheless, the Brazilian Group 2 strains had no deletion in C23L. The deletion site is highlighted with a red box.

**Figure 6:**
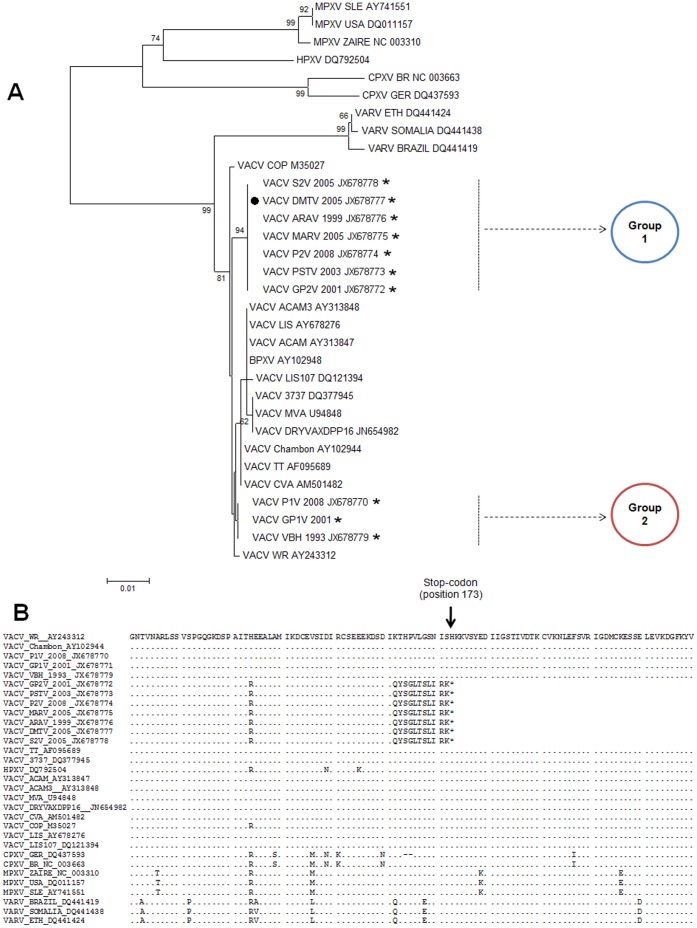
Phylogenetic analysis and sequence alignments of C23L coding sequences. (A) Phylogenetic analysis of C23Lsequences show the grouping of the Brazilian VACV strains into two different branches (Groups 1 and 2), which do not cluster directly with the vaccine strains (vaccine strains: those with no asterisk; and VACV-WR, a laboratorial strain). The DMTV-2005 isolate was clustered with Group 1 as observed with A56R and B5R analyses. (B) Amino acid sequence alignments of the C23L. The stop-codon at position 173 is generated by a ten-nucleotide deletion and highlighted with an arrow. This genetic marker is shared by the DMTV-2005 isolate and other Brazilian VACV strains, such as MARV and GP2V (Group 1), whereas the ten-nucleotide deletion is absenting the GP1V, VBH and P1V strains (Group 2).

### The VACV Outbreak and Viral Isolation

In 2005, a bovine VACV outbreak was reported by IMA (Instituto Mineiro de Agropecuária – IMA), a Brazilian veterinary surveillance institution, in the rural region of Resplendor County, Minas Gerais State, Brazil (Figure 1A). This region is characterized by the presence of several small rural properties, where cattle are kept for milk and meat production. During this outbreak, several animals and farm workers from neighboring properties presented exanthematous lesions similar to those reported during other Brazilian bovine VACV outbreaks (Figure 1B). The origin of this outbreak is unknown, but workers were most likely infected while milking infected animals and spread the virus by direct contact with healthy animals at the other properties. Clinical support was given to the infected workers, who took several days off to recover without the need of hospitalization. A farm that was affected during the outbreak was visited, and an epithelial sample (dried scab) from an infected dairy cow was collected with tweezers, kept under refrigeration and sent to our lab for etiological agent identification. All the collection procedures were performed by veterinarians of IMA, following institutional recommendations (http://www.ima.mg.gov.br). The scab was macerated using a homogenizer (Politron, Littau, Switzerland) in PBS, which contained 200 U/mL penicillin, 4 µg/mL amphotericin B and 100 µg/mL gentamicin (0.1 g scab/0.9 mL PBS), and clarified by centrifugation at 2000×g for 3 min. The resulting supernatant was used for diagnostic purposes, viral isolation and molecular and biological assays [20].

To confirm if the etiological agent of the outbreak was an OPV, the sample supernatants were diluted 1∶100 in PBS and used as templates for a nested-PCR that targeted a partial region of C11R (viral growth factor - *vgf*). This gene is widely used as an OPV diagnostic tool in Brazil [24]. The reactions were carried out by adding 2 µL of the template to 18 µL of the PCR reaction mixture that contained 0.4 mM of *vgf* primers, as previously described [24]. The PCR products were electrophoresed in 8%-PAGE gels and silver stained [25].

For viral isolation, 200 µL of the sample was added onto BSC-40 cell monolayers that were grown in a six-well plate and incubated at 37°C for 72 hours or until detection of cytopathic effect (CPE). After CPE observation, the cells were harvested and new BSC-40 cell monolayers were reinoculated for viral amplification. The resulting viruses were purified in a sucrose gradient and titrated as described [26], [27].

### Biological Tests: Virulence in BALB/c Mice and Plaque Phenotype

To evaluate the virulence profile of this new VACV isolate, named VACV DMTV-2005 (DMTV-2005), groups of four-week-old male BALB/c mice were inoculated intranasally with 10 µL of viral suspensions containing 10^6^ PFU (following the rules of Committee of Ethics for Animal Experimentation from UFMG/Brazil – Comitê de Ética em Experimentação Animal - CETEA). VACV-WR and GP1V were used as virulent controls, whereas GP2V was used as a non-virulent control [19], and the mock infected group was inoculated with 10 µL of PBS. Preceding the viral inoculation, the animals were anesthetized by intraperitoneal injection of ketamine and xylazine (3.2 mg and 0.16 mg/mice in 0.9% PBS, respectively). After inoculation, the mice were housed in filter-top microisolator cages and provided with commercial mouse feed and water *ad libitum*. The animals were kept under observation for 14 days, and clinical signs of infection were recorded daily.

For plaque phenotype assays, BSC-40 cell monolayers at 90–95% confluency were infected with a MOI of 0,01 of the DMTV-2005 isolate, GP1V (as a large-plaque control) and GP2V strains (as a small-plaque control). The VACV-WR strain and PBS were used as additional controls (data not shown). Forty-eight hours after infection, the cells were fixed with paraformaldehyde and stained with crystal violet for plaque size analysis.

### Molecular Characterization

For molecular characterization, viral genes such as C11R (viral growth factor), H5R (morphogenesis-related), B5R (type-I membrane glycoprotein), A56R (hemagglutinin) and C23L (chemokine-binding protein) were amplified and sequenced for phylogenetic analysis. The criteria for these gene selections were as follows: (1st) conserved (C11R and H5R) and non-conserved genes (B5R, A56R and C23L) to conduct phylogenetic studies; (2nd) genes that elucidate the genetic dichotomy among Brazilian VACV strains (B5R, A56R and C23L); (3rd) and established novel genetic markers of Brazilian strains groups (C23L). A56R, B5R, C23L and C11R were amplified with primers as described elsewhere [11], [20], [28]. The H5R primer pair used in this study was as follows: H5R F: ATTATCGCGATATCCGTTAA and H5R R: AGAGTTTACCATCTTTAT.

The chemistry and thermal conditions of these PCR reactions were similar to those used for the *vgf* nested-PCR; however, the annealing temperatures of the specific primers were changed. The PCR fragments obtained from this study were sequenced in both orientations and in triplicates (Mega-BACE 1000 sequencer) (GE Healthcare, Buckinghamshire, UK). The sequences were aligned with previously published OPV sequences from GenBank using the ClustalW method, and the alignments were checked manually with the MEGA version 4.0 software (Arizona State University, Phoenix, AZ, USA). Accession numbers for the analyzed sequences can be found in their respective figures. Maximum likehood trees were reconstructed using different datasets containing sequences from the genes listed above, using MEGA 4.0. Using ModelTest, server [29] the nucleotide substitution model of Tamura 1992 was selected as the best one fitting the data. Rates of variation among sites were estimated for each dataset and two discrete Gamma categories were used to model evolutionary rate differences among sites and the reliability of branching patterns was tested through 1000 bootstrap sampling.

## Results and Discussion

Typical pox-like CPEs were observed in BSC-40 cells that were inoculated with supernatants from the exanthematic clinical samples, such as cell-cell dissociation and migration due to alterations in microtubules, actin and intermediate filaments that culminated in plaque formation in the cell monolayer. Both samples that were collected from infected dairy cows and calves induced CPEs. No cellular changes were observed in the BSC-40 monolayer that was inoculated with PBS. In parallel, these samples were also analyzed with a nested-PCR assay that targets C11R [24] which resulted in the amplification of OPV-specific fragments that were also present in the VACV-WR positive control.

After amplifying and purifying DMTV-2005, plaque phenotype assays and virulence tests in BALB/c mice were performed to evaluate the biological cluster of this new isolate. Previously, Brazilian OPV isolates have been clustered into two distinct groups, Group 1 and Group 2. In addition to genetic differences, Group 1 was shown to be non-virulent in an *in vivo* model using BALB/c mice and to produce small plaques in plaque assays; Group 2 is virulent and produces larger plaques [12], [14], [19]. When BALB/c mice were infected with 10^6^PFU of DMTV-2005 isolate or other VACV strains, we observed that DMTV-2005 behaves like the non-virulent group of VACV Brazilian isolates. DMTV-2005-infected mice did not lose weight over time and survived until the end of the experiment (14 days post-infection – d.p.i.), similar to GP2V-infected mice. In contrast, all animals infected with VACV-WR or GP1V lost weight from the second day after infection and died by day eight or nine, respectively (Figure 2A and 2B). Compared with GP1V and GP2V in plaque assays, DMTV-2005 plaques were small and similar to those formed by GP2V (Figure 2C), which provides additional supporting evidence to classify VACV DMTV-2005 as a member of the non-virulent group. As expected, VACV-WR produced large plaques (data not shown) similar to those associated with GP1V (Figure 2C).

For phylogenetic analysis, five different OPV genes were selected and amplified by PCR using DMTV-2005 as the template, and the resulting amplicons were sequenced. The C11R and H5R sequences of DMTV-2005 indicate that the isolate clusters with other VACV strains (Figures 3A and 3B). Due to the conservation of the nucleotide sequences, C11R and H5R can serve as genetic markers for OPV species identification; however, they provide limited information for VACV sub-cluster analysis. Phylogenetic analyses of B5R and A56R sequences clustered DMTV-2005 with the non-virulent Brazilian isolates (Group 1), confirming our biological data (Figure 4A and B). Moreover, the position of DMTV-2005 on the phylogenetic trees and specific nucleotide substitutions in both B5R and A56R suggest that, although the DMTV-2005 isolate is VACV-related, it is distinct from the vaccine strains (Figure 4, VACV strains with no asterisks), like other Brazilian VACV isolates.

Although A56R and B5R have been validated as specific Brazilian VACV molecular markers, we decided to investigate C23L as another hypothetical gene that may elucidate the Brazilian VACV dichotomy, depending on its variability in other OPV genomes. Analysis of the C23L gene sequence, based on VACV-WR annotation, revealed the presence of a ten-nucleotide deletion (Figure 5), resulting in a frameshift mutation that creates a stop-codon at position 173 (Figure 6B). To analyze the presence of this particular deletion in other Brazilian VACV strains, we amplified and sequenced C23L from strains of Group 1 and Group 2. Remarkably, only the Group 1 isolates had the ten-nucleotide deletion in C23L, including DMTV-2005, in contrast with the C23L of Group 2 isolates. The phylogenetic tree generated from the C23L sequences demonstrates that this gene, along with A56R and B5R, can be used to segregate Brazilian VACV into Group 1 or 2 (Figure 6A). All sequences obtained in this study were deposited in GenBank: JX678770, JX678771, JX678772, JX678773, JX678774, JX678775, JX678776, JX678777, JX678778, JX678779, JX678780, JX678781 and JX678782.

In some *Orthopoxvirus* species C23L encodes for a soluble chemokine-binding viral protein that influences virulence *in vivo*, because C23L knockout *Rabbitpox virus*-infected animals had an increased influx of extravasating leukocytes in the deep dermis during the early phases of infection [30]. By binding certain host chemokines with high affinity, C23L blocks the chemokine interactions with their cellular receptors, abolishing cell transduction pathways and, consequently, inhibiting the chemokine biological activities. Analyses of genomic databases indicate that VACV-WR and others VACV strains may present an upstream start-codon, which would reflect in a distinct coding-frame (unpublish data); as we compared VACV-BR with the prototype VACV-WR and others, our data indicated that VACV-BR Group 1 isolates are not able to produce whole C23L protein, as VACV-WR and VACV-BR Group 2 isolates. However, we believe that new studies are necessary to reveal C23L expression profile and possible alternative transcription frames.

There is no analog to C23L in OPV hosts, so it is unlikely that C23L has been acquired by horizontal transfer [30], [31], [32]. Even though there is no supporting biological data concerning C23L importance to Brazilian VACV virulence, we can speculate that loss of function due to a premature stop-codon in the C23L open reading frame of Group 1 Brazilian VACV isolates may be a factor that contributes to decreased virulence of this group. The ten-nucleotide deletion in C23L can also serve as a marker for non-virulent Brazilian VACV viruses and may be used to characterize new isolates or exploited in differential PCR-based screening with primers that either bind to the deleted sequence or not (Kroon, unpublished data). C23L has already been incorporated into diagnostic assays; oligonucleotide probes designed to detect the C23L sequence have been included in the microarrays for OPV detection [33], [34]. C23L has also been used successfully for OPV phylogenetic analysis, dividing twelve European CPXV isolates into two major clades and up to five distinct monophyletic clusters [35].

We conclude that DMTV-2005 is a new Group 1 Brazilian VACV strain (non-mice virulent) that was isolated from an outbreak of bovine vaccinia in the southeast region of Brazil. Farming and dairy production are important activities in this region that were negatively impacted by the bovine vaccinia outbreaks. Our analyses showed that the C23L sequences of several Brazilian VACV isolates in the non-virulent group share a unique ten-nucleotide deletion. This deletion may cause a frameshift mutation, which would result in a stop-codon that may lead to a truncated C23L protein; although new studies are required focusing the C23L promoter and alternative transcription frames, this deletion can be considered to be a putative genetic marker for non-virulent Brazilian VACV isolates and may be used for the detection and molecular characterization of new isolates.

## References

[1] Damon I (2007) Poxviridae and their replication. Fields Virology. New York: Raven Press. 2079–2081.

[2] Fenner F, Henderson DA, Arita I, Jezek A, Ladnyi I D (1988) Smallpox and its eradication. Geneva: World Health Organization Press. 49 p.

[3] International Committee on Taxonomy of Viruses (ICTV) (2009). Available: http://www.ncbi.nlm.nih.gov/ICTVdb/. Accessed: 2012 May 14.

[4] Heymann DL, Aylward RB (2006) Mass vaccination: when and why. Curr Top MicrobiolImmunol 304: 1–16. 10.1007/3-540-36583-4_1 16989261

[5] Haenssle HA, Kiessling J, Kempf VA, Fuchs T, Neumann C, et al (2006) Orthopoxvirus infection transmitted by a domestic cat. J Am AcadDermatol 54: S1–4. 10.1016/j.jaad.2005.09.040 16427982

[6] Reynolds MG, Yorita KL, Kuehnert MJ, Davidson WB, Huhn GD, et al (2006) Clinical manifestations of human monkeypox influenced by route of infection. J Infect Dis 194: 773–780. 16941343 10.1086/505880

[7] Damaso CR, Esposito JJ, Condit RC, Moussatche N (2000) An emergent poxvirus from humans and cattle in Rio de Janeiro State: Cantagalo virus may derive from Brazilian smallpox vaccine. Virology 277: 439–449. 11080491 10.1006/viro.2000.0603

[8] de Souza GT, da Fonseca FG, Marques JT, Nogueira ML, Mendes LC, et al (2003) Aracatuba virus: a vaccinialike virus associated with infection in humans and cattle. Emerg Infect Dis 9: 155–160. 12603984 10.3201/eid0902.020244 PMC2901946

[9] Singh RK, Hosamani M, Balamurugan V, Bhanuprakash V, Rasool TJ, et al (2007) Buffalopox: an emerging and re-emerging zoonosis. Anim Health Res Rev 8: 105–114. 17692147 10.1017/S1466252307001259

[10] Trindade GS, Emerson GL, Carroll DS, Kroon EG, Damon IK (2007) Brazilian vaccinia viruses and their origins. Emerg Infect Dis 13: 965–972. 18214166 10.3201/eid1307.061404 PMC2878226

[11] Drumond BP, Leite JA, da Fonseca FG, Bonjardim CA, Ferreira PC, et al (2008) Brazilian Vaccinia virus strains are genetically divergent and differ from the Lister vaccine strain. Microbes Infect 10: 185–197. 18248758 10.1016/j.micinf.2007.11.005

[12] Abrahão JS, Guedes MI, Trindade GS, Fonseca FG, Campos RK, et al (2009) One more piece in the VACV ecological puzzle: could peridomestic rodents be the link between wildlife and bovine vaccinia outbreaks in Brazil? PLoS ONE 4: 7421–7428. 10.1371/journal.pone.0007428 PMC2758550 19838293

[13] Brum MCS, dos Anjos BL, Nogueira CEW, Amaral LA, Weiblen R, et al (2010) An outbreak of orthopoxvirus-associated disease in horses in southern Brazil. J Vet Diagn Invest22: 143–147. 10.1177/104063871002200132 20093706

[14] Campos RK, Brum MCS, Nogueira CEW, Drumond BP, Alves PA, et al (2010) Assessing the variability of Brazilian Vaccinia virus isolates from a horse exanthematic lesion: coinfection with distinct viruses. ArchVirol 156: 275–283. 10.1007/s00705-010-0857-z 21080203

[15] Leite JA, Drumond BP, Trindade GS, Lobato ZIP, da Fonseca FG, et al (2005) Passatempo virus, a vaccinia virus strain, Brazil. Emerg Infect Dis 11: 1935–8. 16485483 10.3201/eid1112.050773 PMC3367646

[16] Lobato ZIP, Trindade GS, Frois MCM, Ribeiro EBT, Dias GRC, et al (2005) Outbreak of exanthemal disease caused by vaccínia virus in human and cattle in Zona da Mata region, Minas Gerais [in Portuguese]. Arq Bras Med Vet Zootec. 57: 423–429.

[17] Megid J, Appolinário CM, Langoni H, Pituco EM, Okuda LH (2008) Vaccinia virus in humans and cattle in southwest region of Sao Paulo state, Brazil. Am J Trop Med Hyg 79: 647–651. 18981497

[18] Trindade GS, Guedes MI, Drumond BP, Mota E, Abrahão JS, et al (2009) Zoonotic vaccinia virus: clinical and immunological characteristics in a naturally infected patient. Clin Infect Dis 48: 37–40. 10.1086/595856 19115976

[19] Ferreira JM, Drumond BP, Guedes MI, Pascoal-Xavier MA, Almeida-Leite CM, et al (2008) Virulence in murine model shows the existence of two distinct populations of Brazilian Vaccinia virus strains. PLoS ONE 3: 3038–3043. 10.1371/journal.pone.0003043 PMC2518622 18725979

[20] Abrahão JS, Drumond BP, Trindade GS, da Silva-Fernandes AT, Ferreira JM, et al (2010) Rapid detection of Orthopoxvirus by semi-nested PCR directly from clinical specimens: a useful alternative for routine laboratories. J Med Virol 82: 692–699. 20166167 10.1002/jmv.21617

[21] Medaglia ML, Pessoa LC, Sales ER, Freitas TR, Damaso CR (2009) Spread of cantagalo virus to northern Brazil. Emerg Infect Dis 15: 1142–3. 19624947 10.3201/eid1507.081702 PMC2744230

[22] Leite JA, Drumond BP, Trindade GS, Bonjardim CA, Ferreira PCP, et al (2007) Brazilian Vaccinia virus strains show genetic polymorphism at the ati gene. Virus Genes 35: 531–539. 17671837 10.1007/s11262-007-0133-9

[23] Trindade GS, Lobato ZIP, Drumond BP, Leite JA, Trigueiro RC, et al (2006) Short report: Isolation of two vaccinia virus strains from a single bovine vaccínia outbreak in rural area from Brazil: Implications on the emergence of zoonotic orthopoxviruses. Am J TropMedHyg 75: 486–90. 16968926

[24] Abrahão JS, Lima LS, Assis FL, Alves PA, Silva-Fernandes AT, et al.. (2009) Nested-multiplex PCR detection of Orthopoxvirus and Parapoxvirus directly from exanthematic clinical samples. Virol J 6: 140, 1–5. 10.1186/1743-422X-6-140 PMC2749831 19747382

[25] Sambrook J, Russell DW (2001) Molecular Cloning: A Laboratory Manual, 3rd Edn. Cold Spring Harbor, New York.

[26] Joklik WK (1962) The purification of four strains of poxvirus. Virology 18: 9–18. 14036977 10.1016/0042-6822(62)90172-1

[27] Campos MAS, Kroon EG (1993) Critical period for reversible block of vaccinia virus replication. Rev Braz Microbiol 24: 104–110.

[28] Ropp SL, Qi J, Janice CK, Massung RF, Esposito JJ (1995) PCR Strategy for Identification and Differentiation of Smallpox and Other Orthopoxviruses. J ClinMicrobiol 33: 2069–2076. 10.1128/jcm.33.8.2069-2076.1995 PMC228337 7559950

[29] David Posada (2006) ModelTest Server: a web-based tool for the statistical selection of models of nucleotide substitution online. Nucleic Acids Res. 34(Web Server issue): W700–W703. 10.1093/nar/gkl042 PMC1538795 16845102

[30] Graham KA, Lalani AS, Macen JL, Ness TL, Barry M, et al (1997) The T1/35 kDa family of poxvirus-secreted proteins bind chemokines and modulate leukocyte influx into virus-infected tissues. Virology 229: 12–24. 9123853 10.1006/viro.1996.8423

[31] Alcamí A, Symons JA, Collins PD, Williams TJ, Smith GL (1998) Blockade of chemokine activity by a soluble chemokine binding protein from vaccinia virus. J Immunol 160: 624–633. 9551896

[32] Smith CA, Smith TD, Smolak PJ, Friend D, Hagen H, et al (1997) Poxvirus genomes encode a secreted, soluble protein that preferentially inhibits beta chemokine activity yet lacks sequence homology to known chemokine receptors. Virology 236: 316–327. 9325239 10.1006/viro.1997.8730

[33] Laassri M, Chizhikov V, Mikheev M, Shchelkunov S, Chumakov K (2003) Detection and discrimination of orthopoxviruses using microarrays of immobilized oligonucleotides. J Virol Methods 112: 67–78. 12951214 10.1016/S0166-0934(03)00193-9 PMC9533938

[34] Ryabinin VA, Shundrin LA, Kostina EB, Laassri M, Chizhikov V, et al (2006) Microarray assay for detection and discrimination of Orthopoxvirus species. J Med Virol 78: 1325–1340. 16927285 10.1002/jmv.20698

[35] Carroll DS, Emerson GL, Li Y, Sammons S, Olson V, et al (2011) Chasing Jenner’s vaccine: revisiting cowpox virus classification. PLoS ONE 6: 23080–23086. 10.1371/journal.pone.0023086 PMC3152555 21858000

